# *In vitro* application of ribonucleases: comparison of the effects on mRNA and miRNA stability

**DOI:** 10.1186/s13104-015-1114-z

**Published:** 2015-04-22

**Authors:** Arian Aryani, Bernd Denecke

**Affiliations:** Interdisciplinary Center for Clinical Research Aachen (IZKF Aachen), RWTH Aachen University, Pauwelsstrasse 30, Aachen, Germany

**Keywords:** mRNA, miRNA, Ribosomal RNA, Ribonuclease, RNA stability, RNA integrity

## Abstract

**Background:**

MicroRNA has become important in a wide range of research interests. Due to the increasing number of known microRNAs, these molecules are likely to be increasingly seen as a new class of biomarkers. This is driven by the fact that microRNAs are relatively stable when circulating in the plasma. Despite extensive analysis of mechanisms involved in microRNA processing, relatively little is known about the *in vitro* decay of microRNAs under defined conditions or about the relative stabilities of mRNAs and microRNAs.

**Methods:**

In this *in vitro* study, equal amounts of total RNA of identical RNA pools were treated with different ribonucleases under defined conditions. Degradation of total RNA was assessed using microfluidic analysis mainly based on ribosomal RNA. To evaluate the influence of the specific RNases on the different classes of RNA (ribosomal RNA, mRNA, miRNA) ribosomal RNA as well as a pattern of specific mRNAs and miRNAs was quantified using RT-qPCR assays. By comparison to the untreated control sample the ribonuclease-specific degradation grade depending on the RNA class was determined.

**Results:**

In the present *in vitro* study we have investigated the stabilities of mRNA and microRNA with respect to the influence of ribonucleases used in laboratory practice. Total RNA was treated with specific ribonucleases and the decay of different kinds of RNA was analysed by RT-qPCR and miniaturized gel electrophoresis. In addition, we have examined whether the integrity observed for ribosomal RNA is applicable to microRNA and mRNA. Depending on the kind of ribonuclease used, our results demonstrated a higher stability of microRNA relative to mRNA and a limitation of the relevance of ribosomal RNA integrity to the integrity of other RNA groups.

**Conclusion:**

Our results suggest that the degradation status of ribosomal RNA is not always applicable to mRNA and microRNA. In fact, the stabilities of these RNA classes to exposure to ribonucleases are independent from each other, with microRNA being more stable than mRNA. The relative stability of microRNAs supports their potential and further development as biomarkers in a range of applications.

## Background

MicroRNAs (miRNAs) are small, non-coding RNAs which are encoded within the genome. The primary transcript, pri-miRNA, contains one or more hairpin structures and is cleaved to pre-miRNA [1,2]. Upon export to the cytoplasm, dicer cleaves the pre-miRNA to mature miRNA, which usually has a length of about 22 ribonucleotides [1,3,4].

Previous studies have suggested that mRNA stability is an important control point for gene expression regulation [5]. The mature miRNA plays a key role in the regulation of their specific target genes [6]. The regulatory function of miRNA is differentiated into two distinct mechanisms: the inhibition of target gene expression (i) by inducing the degradation of the target mRNA [4] and/or (ii) by inhibition of target gene translation [7]. Partial complementary base pairing causes translational inhibition whereas perfect base pairing causes degradation of the complementary target mRNAs. It has long been assumed that translational inhibition is the dominant mechanism, however, recent studies have shown that the degradation of target mRNA plays the major role in the inhibition of protein expression [8].

A widespread interest has developed in small non-coding RNAs for cancer research and drug development. In particular, miRNAs appear to play a critical role in tumorigenesis and are thus promising candidates as potential therapeutic targets or as biomarkers [9,10]. Moreover, miRNAs are able to regulate the expression of multiple genes and hence affect disease pathways at multiple points [10]. Therefore, miRNAs are promising tools in diagnostic and therapeutic applications in a wide range of specializations, including cancer research [11], cardiovascular disease [12], organ transplantation [13] and rheumatoid arthritis [14].

Although miRNAs comprise an important class molecules used to uncover regulatory mechanisms in the cell, nearly nothing is known about their decay in comparison to mRNA under defined *in vitro* conditions. For this reason, we decided to enhance general knowledge about the stability of mRNA and miRNA in response to ribonucleases commonly used in routine laboratory practice. We were interested in finding out how a hypothetical contamination with ribonuclease might affect mRNA and miRNA stability. Furthermore, we investigated possible differences in the stability of different classes of RNA (i.e. miRNA, mRNA or ribosomal RNA). For this purpose, total RNA was treated *in vitro* with a range of ribonucleases under identical experimental conditions and their effects were analysed by RT-qPCR.

## Results

### The integrity of ribosomal RNA is affected by treatment with specific ribonucleases

In contrast to mRNA, which represents only 0.5 to 3% of the transcriptome, ribosomal RNA is the most prevalent constituent of total RNA in mammalian cells [15]. For this reason the quality of mRNA is generally extrapolated on the basis of the quality of ribosomal RNA, which can be analysed, for example, with microfluidic analysis (Agilent 2100 Bioanalyzer).

**Figure 1:**
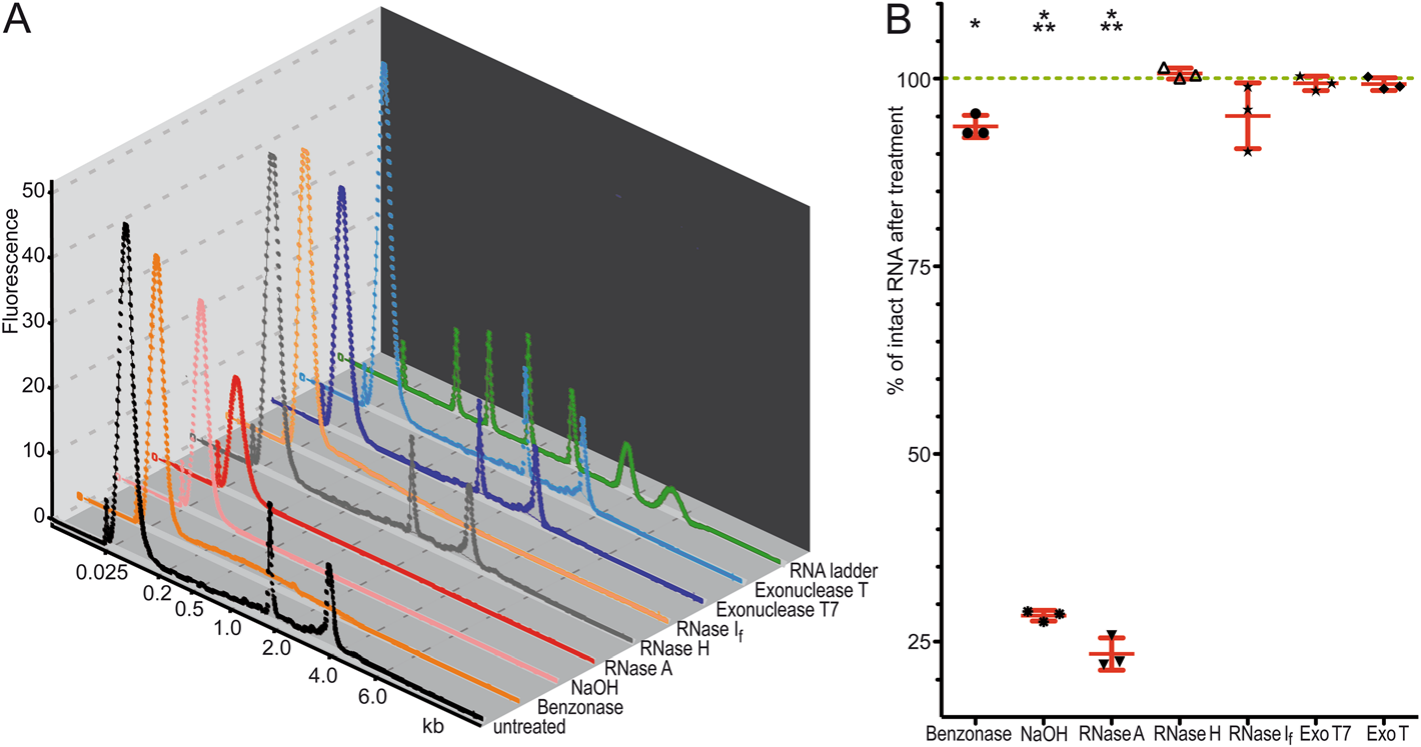
The Stability of ribosomal RNA is affected in a treatment-dependent manner. **(A)** Representative electropherograms of untreated RNA (black) and RNA after treatment with either Benzonase (dark orange), NaOH (pink), RNase A (red), RNase H (grey), RNase I_f_ (bright orange), Exonuclease T_7_ (dark blue) or Exonuclease T (bright blue). Electropherogram of RNA ladder, used for size calculation indicated in the abscissa as kilobases (kb), is illustrated in green. Arbitrary units for fluorescence are given in the ordinate. The untreated control sample represents four peaks: the loading marker at 25 nt, the spiked tRNA at about 80 nt, the 18S and the 28S ribosomal RNA. **(B)** Detection of 18S ribosomal RNA using RT-qPCR. Percentage of intact 18S ribosomal RNA after indicated treatments is illustrated for three independent experiments with mean and SD (in red). The relative percentage of intact RNA in comparison to the untreated RNA is given in the ordinate. The green dotted line represents the 100% intact 18S ribosomal RNA obtained from the untreated RNA. Significances were calculated by the two tailed unpaired Student’s t-test: * = p < 0.05; *** = p < 0.001. ExoT7 = Exonuclease T_7_; ExoT = Exonuclease T.

Representative electropherograms of our samples that were analysed with the RNA6000 Nano labchip are illustrated in Figure 1A. The electropherogram of the untreated sample consists of four peaks: i) the loading marker at 25 nt, ii) the spiked tRNA at about 80 nt, iii) the 18S ribosomal RNA and, iv) the 28S ribosomal RNA. The tRNA was added after the treatments in order to facilitate the precipitation of RNA. It was clear that the 18S and 28S ribosomal RNAs were missing after NaOH treatment. This was expected because NaOH, at the concentration used, induces the hydrolysis of RNA in a manner that is independent of size and sequence. Similar to NaOH, RNase A-treated samples lacked both 18S and 28S peaks. This indicated that both NaOH and RNase A treatments significantly affected ribosomal RNA.

In the case of treatment with benzonase and RNase I_f_, even though the ribosomal peaks were not detectable by this method, the measured absorption was above the base line. This is caused by residues of ribosomal RNA. No specific changes in comparison to the untreated sample were observed in the electropherograms of the samples after treatment with RNase H, Exonuclease T or Exonuclease T_7_.

In order to determine the decay of ribosomal RNA after the treatments with a higher sensitivity, a relative measurement of 18S RNA was performed with RT-qPCR (Figure 1B). The percentage of undigested 18S ribosomal RNA normalised to the untreated control is presented in Figure 1B. It was observed that, similar to the results obtained by electrophoresis after treatment with RNase A or NaOH, a significant reduction of ribosomal RNA was detectable (a reduction of 77% and 66%, respectively). The observed difference in reduction between the two methods was probably caused by the higher sensitivity of the RT-qPCR.

A similar effect could be observed after treatment with benzonase and RNase I_f_. In contrast to a dramatic reduction of ribosomal RNA detected by electrophoresis, the results obtained by RT-qPCR showed only a moderate reduction. In the case of benzonase, this reduction was significant (p = 0.027). For treatment with RNase I_f_, a statistically non-significant trend of reduction was observed.

The treatments with RNase H, Exonucleases T and Exonucleases T_7_ showed no significant changes compared to the untreated sample. This confirmed the result obtained by microfluidic analysis in a quantitative manner.

In the case of RNase I_f_ treatment, comparison of sample electropherograms, which represented the 18S and 28S ribosomal RNAs (Figure 1A), with the results obtained by 18S RT-qPCR (Figure 1B), revealed a discrepancy between these two sets of results. The microfluidic analysis revealed a partial degradation of 18S and 28S following treatment with RNase I_f_ , while the RT-qPCR demonstrated only a partial, non-significant digestion of the 18S ribosomal RNA.

Taken together, these results indicate that, under experimental conditions, RNase A, benzonase, NaOH and RNase I_f_ treatments cause a total or partial decay of ribosomal RNA.

### Detection of mRNA and miRNA are represented by targets with different frequencies

Next we asked if the stability of ribosomal RNA to the various treatments was representative of the stabilities of mRNA and/or miRNA to the same treatments. This knowledge is necessary to assess the relative influence of various treatments on different classes of RNA. For this reason we performed RT-qPCR for different mRNA and miRNA targets in the same samples as used for the ribosomal RNA analyses.

**Figure 2:**
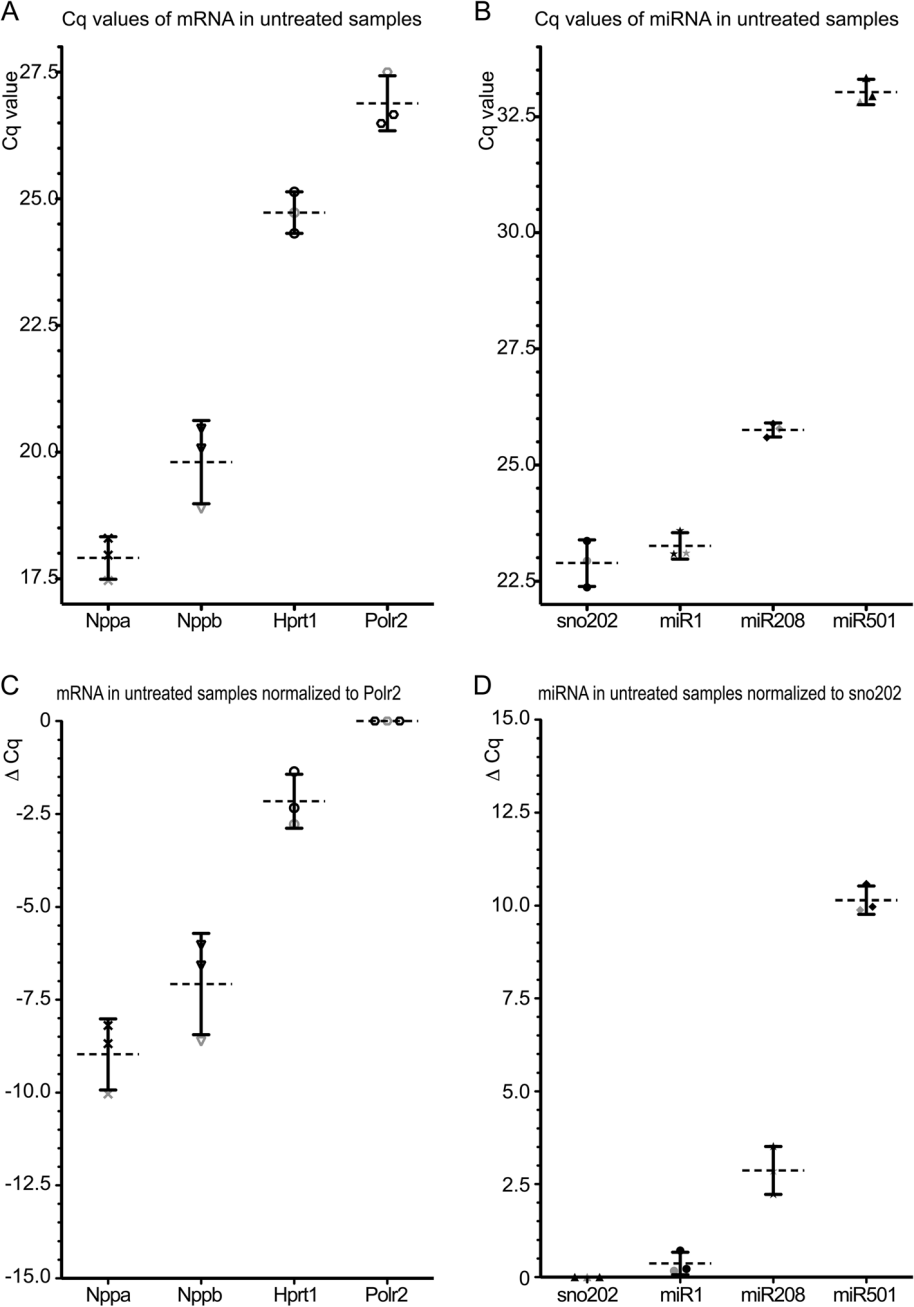
Analysed targets represent a wide range of quantities. The quantification cycle (Cq), which is inversely proportional to the amount of target nucleic acid in the sample, was used to calculate the approximate amount of the targets. RT-qPCR analysis was performed with the untreated RNA isolated from three independent mice hearts (one male - grey, and two female - black). **(A)** Data-points and the mean of the Cq values of Nppa (cross), Nppb (downward-pointing triangle), Hprt (open circle) and Polr2a (hexagon) with the SD of three independent experiments are illustrated. **(B)** Data-points and the mean of the Cq values of Sno202 (closed circle), miR-1 (asterisk), miR-208 (rhombus) and miR-501 (upwards-pointing triangle) with the SD of three independent experiments are illustrated. **(C)** Data-points and the mean of the ΔCq values of Nppa (cross), Nppb (downward-pointing triangle), Hprt (open circle) and Polr2a (hexagon) with the SD of three independent experiments after normalization with the reference mRNA Polr2. **(D)** Data-points and the mean of the ΔCq values of Sno202 (upwards-pointing triangle), miR-1 (closed circle), miR-208 (asterisk) and miR-501 (rhombus) with the SD of three independent experiments after normalization with the reference small RNA sno202.

The mRNA stability was investigated by RT-qPCR for expression of *Hprt* (normally used as a reference gene) [16], *Polr2a* (normally used as a reference gene) [17], *Nppa* (gene expressed in heart muscles) and *Nppb* (gene also expressed in heart muscles) [18]. First, we compared the frequency of the targets analysed in the untreated samples. As illustrated in Figure 2A, Nppa was most highly expressed (Cq = 17.9 ± 0.4 cycles), followed by Nppb (Cq = 19.8 ± 0.8 cycles), Hprt (Cq = 24.7 ± 0.4 cycles) and Polr2a (Cq = 26.9 ± 0.5 cycles).

On the basis of our previous quantitative studies [19], miR-1 and miR-208 (cardiac-specific expressed miRNAs) [20], sno202 (small nuclear RNA 202, often used as a normalization control) [21], and miR-501 [22] were investigated as indicators of miRNA stability. Figure 2B illustrates the Cq values of the miRNA targets in the untreated controls. Sno202 was more highly expressed in heart tissue than the other miRNAs (Cq = 22.9 ± 0.5 cycles), followed by miR-1 (Cq = 23.3 ± 0.3 cycles) and miR-208 (Cq = 25.8 ± 0.2 cycles). For miR-501, the lowest expression was detected (Cq =33.0 ± 0.3 cycles). To determine whether normalization to a reference gene influenced the results, we normalized the mRNA values using Polr2, and the miRNA values using sno202. As illustrated in Figure 2C and D, the distribution of the delta Cq values looked similar to the Cq values without normalization. The relatively high standard deviation for mRNA seems to be caused by the different expression levels between male and females. Such a gender distinction was not visible for the analyzed miRNAs.

For both mRNA and miRNA, the selected targets cover a broad range of expression frequency (Cq 17.9 - 26.9 and Cq 22.9 - 33.0, respectively). This would ensure the uncovering of potential treatment effects depending on the frequency of a specific target RNA. However, a target RNA-dependent frequency was not observed. Consequently, in order to compensate for the different target frequencies (i.e to have the result independent from the original Cq value of each target), we normalized the results to the Cq value of the corresponding untreated sample in our further analyses.

### Ribonucleases do not have the same effect on different classes of RNA

**Figure 3:**
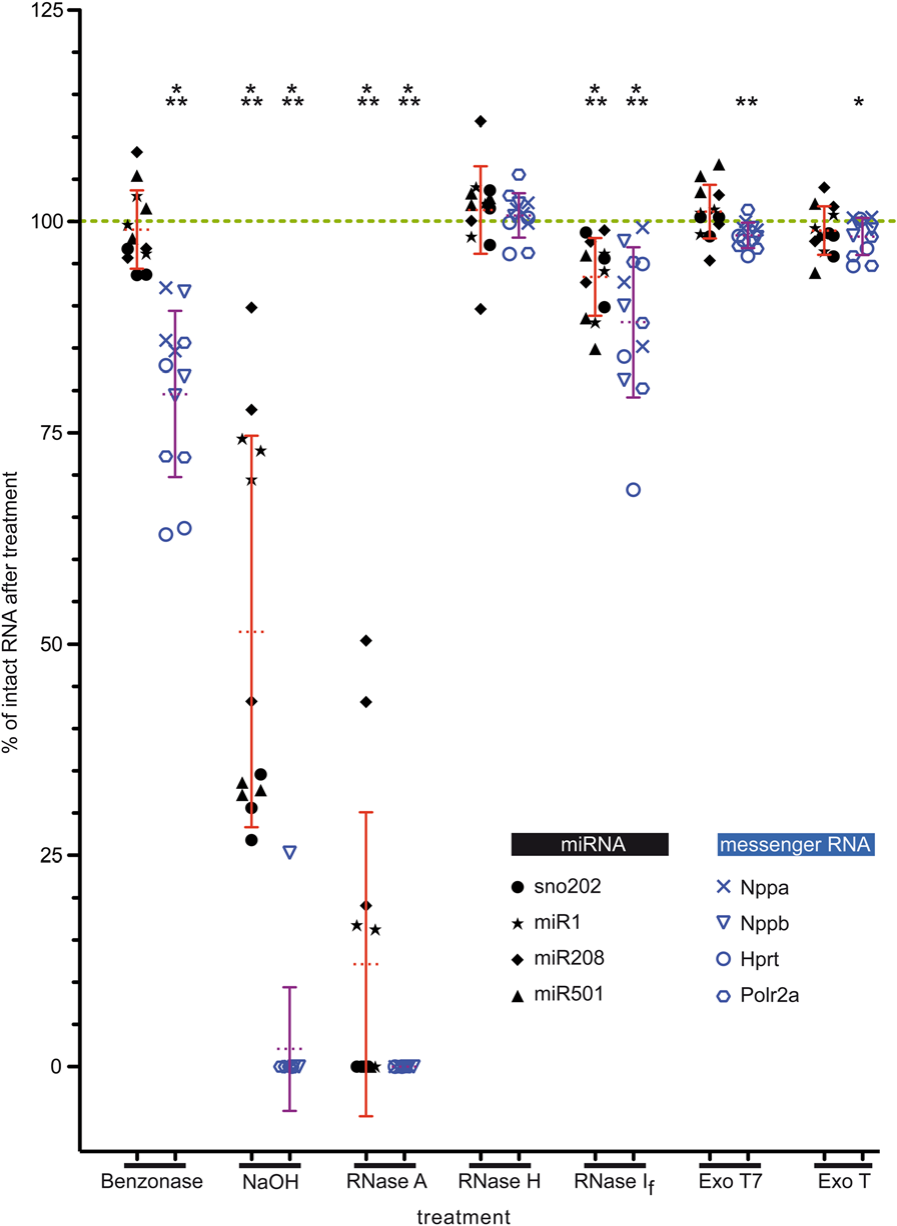
mRNA and miRNA stabilities measured by RT-qPCR are differently influenced depending on the treatment. Percentage of intact mRNA (blue) and miRNA (black) after indicated treatments are illustrated for three independent experiments. Nppa, Nppb, Hprt, and Polr2a were each analysed as targets representative for mRNA and sno202, miR1, miR208, and miR501 each as targets for miRNA. The mean with the SD for all analysed targets of each RNA class is given in violet for mRNA and in red for miRNA. The green dotted line represents the 100% intact RNA obtained from the untreated RNA. The ordinate indicates the relative percentage of intact RNA in comparison to the untreated RNA. Significances were tested by the two tailed unpaired Student’s t-test: * = p < 0.05; ** = p < 0.01; *** = p < 0.001. ExoT_7_ = Exonuclease T_7_; ExoT = Exonuclease T.

Next we considered whether the stability of mRNA and miRNA was the same after the different treatments. We measured the relative amount of intact mRNA/miRNA depending on the different treatments for the selected targets. In Figure 3, the relative amount of the normalised intact targets is shown as a percentage of untreated control. For every treatment, the percentages of intact targets for three independent experiments are represented for mRNA and miRNA. In addition, the sample mean and standard deviation are shown. The results could be divided into five categories.

i) The first category represented the significant decay of mRNA and miRNA, including treatments with NaOH and RNase A. The RT-qPCR results for the mRNA targets suggested the total decay of mRNAs after the treatment with NaOH or RNase A. Similarly, after the NaOH treatment, the average of undigested miRNAs was reduced by 48.5%. The reduction for RNase A-treated samples was more prominent (i.e. 87.9%). Therefore, these treatments resulted in a severe decay of miRNAs. These results are consistent with those obtained by microfluidic analysis and by RT-qPCR for the ribosomal RNA, which also showed a significant amount of decay.
ii) The second category consisted of Exonuclease T and Exonuclease T_7_ treatments which resulted in the digestion of mRNAs but not of miRNAs. Although the reduction of undigested mRNA was only by about 2% in both cases, it was found to be significant. As in the case of miRNA, ribosomal RNA did not show any changes in RNA integrity or in 18S levels by RT-qPCR (Figure 1).
iii) The third category was represented only by RNase I_f_. The amounts of intact mRNA and miRNA were both moderately, but significantly reduced (11.9% for mRNA and 6.6% for miRNA). This indicated that RNase I_f_ digested miRNA as well as mRNA, but in a moderate manner. Digestion of ribosomal RNA could also be observed in the electropherogram of the microfluidic analysis. However, the partial digestion of 18S RNA represented by RT-qPCR was not significant (Figure 1B).
iv) RNase H represented the fourth category, which caused no effect on mRNA or miRNA or ribosomal RNA.
v) Benzonase represented the last category. After treatment with benzonase, a significant reduction of intact mRNAs was observed (20.4%). Contrary to this result, no reduction was seen for miRNA. The partial digestion of ribosomal RNA by benzonase as analysed by microfluidic analysis and 18S RT-qPCR was in line with the results of those for mRNA. Thus, benzonase precisely affected ribosomal RNA and mRNA, but had no effect on miRNA.

The results of our study indicate that, depending on the kind of treatment (i.e. ribonucleases), mRNA and miRNA demonstrate different stabilities. Notably, in comparison to mRNA, miRNA frequently presented a higher level of stability. Taken together, our results indicate that NaOH and all analysed ribonucleases (except RNase H) affected mRNA and resulted in the decay of mRNA. On the one hand, benzonase, Exonuclease T_7_ and Exonuclease T significantly affected mRNA but had no significant effect on decay of miRNA. On the other hand, treatments with NaOH, RNase A and RNase I_f_ also affected miRNA. In addition, after specific treatments of total RNA, microfluidic analyses revealed that ribosomal RNA was degraded whereas miRNA, as assessed by RT-qPCR, exhibited no degradation relative to the untreated sample. Hence, we conclude that there is not always a direct correlation between the integrity of ribosomal RNA and that of mRNA or of miRNA.

## Discussion

Significant amounts of miRNAs, which are remarkably stable, can be detected in all biological fluids. This has favoured the use of miRNAs as a new class of biomarkers. It is assumed that the high stability of miRNAs in biological fluids is based on the fact that they are protected from endogenous RNase activity by being packed inside exosomes or that they are protected via association with an AGO protein complex [23,24]. The purpose of the present study was to investigate the stability of different classes of RNA (miRNA, mRNA, and rRNA) against common sources of contamination in a molecular biology laboratory.

In eukaryotes, the most common method used to assess the integrity of RNA is the measurement of the 28S to 18S ribosomal RNA where the ratio for intact RNA is 2:1. Previously, this assessment was done by running an RNA aliquot on a denaturing agarose gel stained with ethidium bromide. Other nucleic acid stains such as SYBR Gold increase the sensitivity, also enabling the detection of lower amounts loaded on the gel. In the meantime, RNA integrity is assessed using microfluidic analysis (e.g. Agilent 2100 Bioanalyzer, AdvanceCE™ Nucleic Acids Analyzer, or BioRad Experion). By contrast, absorbance or fluorescent dye-based procedures are useful for the quantification of RNA and, if necessary, for the purity. These techniques are, however, unsuitable for the determination of RNA integrity. The assessment of mRNA or miRNA integrity in total RNA, independent of ribosomal RNA, is proving extremely difficult and mostly includes RT-qPCR. Although there are also microfluidic analysis assays for small RNAs (e.g. Agilent small RNA assay) these assays are influenced by total RNA integrity [25]. For these reasons, microfluidic analysis of total RNA is widely used for the assessment of total RNA integrity. The integrity of mRNA and miRNA is normally extrapolated from the measured ribosomal integrity.

Our results show that the degradation status of ribosomal RNA is not, in all cases, applicable for the degradation status of mRNA and miRNA. In addition, a class-dependent stability for mRNA, miRNA and ribosomal RNA was detected in presence of specific ribonucleases. The class-dependent stability was supported by the observation that the analysed - RNA class-specific - targets showed, more or less, the same stability. In general, miRNAs revealed more stability than mRNAs to exposure to ribonucleases and NaOH. This indicates miRNAs to be good, candidate biomarkers.

Apart from the important regulatory effect of miRNA on mRNA stability and/or translation [8], the dysregulation of miRNAs can function as a biomarker for use in cancer diagnosis and for many other diseases [10-14]. In this context it is important to know if the observed dysregulation is due to the regulation process or due to the differential stabilities of mRNA and miRNA.

Differential stabilities may have extensive consequences for analysis of the observed data. For instance, in the case of detection of increased levels of miRNA and a decreased level of the corresponding target(s) mRNA, the conclusion would be that the putative down-regulation of mRNA is due to the regulatory effect of miRNA. However, the lower stability of mRNA would not have been considered. Normalization to endogenous reference genes is a useful procedure to correct for sample-to-sample variations in RT-qPCR efficiency and errors in sample quantification. The expression is estimated relative to the levels of the (invariant!) endogenous references [26]. However, this corrective action does not take effect between mRNA and miRNA, especially if these are degraded with different efficiencies.

Furthermore, we have performed the experiments with ribonuclesases that are commonly used in a molecular biology laboratory. These ribonucleases are frequently connected with a high possibility of reaction contamination. To our knowledge, up to the time of this study, there is no information available regarding the *in vitro* effect of the analysed ribonucleases on classes of RNA.

In a previous study it was shown that, even in presumed heat-degraded total RNA, the robust stability of miRNA enabled its quantification. In contrast, the measurement was no longer possible for mRNA [27]. This study has also shown that the measured RNA integrity does not adversely affect the accuracy of RT-qPCR measurements of miRNA, although it significantly affects the measurement of mRNA [27]. Thus, degradation by heat results in major effects on mRNA in comparison to miRNA, and miRNA represents a relatively stable form. Another study also indicated that miRNA possess distinct stability. This stability depends on factors such as 7 nucleotides on 3′ terminal or the specific sequences within a miRNA that make the miRNA a target for ribonucleases. Also, some miRNAs are associated with components of RISC such as Ago 2, which also influence the miRNA stability [28]. However, in this study we were more interested in comparing the stability of mRNA and miRNA after treatment with commonly used ribonucleases, being the main source of contamination in routine molecular biology laboratories. This important aspect may affect the assessment of RNA quality and thus subsequent results of experiments. Precisely, we have shown that degradation by different ribonucleases is RNA class-dependent. Our results also confirmed that RNA integrity would not consistently affect the assessment of the degradation of miRNA.

RNase A is an endoribonuclease that attacks single-stranded RNA 3′ to pyrimidine residues and cleaves the phosphate linkage to the adjacent nucleotide [29,30]. This RNase is one of the major contamination sources in the molecular biology laboratory. NaOH also catalyses the breaking of the phosphate backbone in RNA. As expected, both treatments (RNase A and NaOH) significantly decayed all three analysed classes of RNA.

Moreover, RNase I_f_ is one of the few known ribonucleases which cleaves the phosphodiester bond between any two nucleotides [31]. It is a single strand specific endoribonuclease. It was reported that RNase I_f_ favours the degradation of small RNA oligonucleotides but also degrades long RNA polymers very slowly [32]. This earlier finding would explain why all three examined classes of RNA decayed, however, the observable decay for ribosomal RNA was not found to be significant.

RNase H cleaves RNA *via* a hydrolytic mechanism in DNA/RNA duplexes [33]. This was confirmed in our experiments because no decay was observed in any class of RNA.

It was reported that Exonuclease T_7_ functions similar to RNase H on double-stranded DNA/RNA hybrids. It specifically degrades the RNA region of poly(T)-poly(A) hybrid polymers from 5′ terminus [34]. On the other hand, Exonuclease T is a single stranded RNA or DNA specific nuclease and removes nucleotides in the 3′ to 5′ direction. Exonuclease T also acts on single-stranded RNA in a sequence-specific manner. Both Exonucleases do not decay ribosomal RNA and miRNA but they do decay mRNA. The small but significant decay of mRNA by Exonuclease T might be due to sequence specificity of the enzyme, which has to be clarified in future studies. The small decay obtained for mRNA after treatment with Exonuclease T_7_ might have resulted from undefined cleavage, which is favoured for mRNA.

Benzonase degrades all forms of DNA and RNA and is almost capable of cleavage at all positions along a nucleic acid chain. However, a sequence dependency was demonstrated. A favourable digestion of DNA and RNA in a double-stranded G- and C-rich sequence has been reported [29,35]. The reason for the observed missing decay of miRNA could be explained by the small size of miRNA. Either small size RNAs, in general, do not act as a target for benzonase or the probability is very low that the target sequence is represented in the miRNA.

In the present study we have used RNA from male and female mouse hearts. This is important to show that results are consistent across both genders. Also we have generated the results in three independent experiments to prove the reproducibility of the results and to highlight the prominent effect of the ribonucleases. However, this study can be expanded by providing other RNA samples or using other treatments. Genome-wide analyses of the treatment-mediated effects on the different classes of RNA are possible by using microarray technology. Using this method all targets would be amenable to support the observed RNA class-dependent stability and thus to generalize our findings. One limitation of our study was to focus solely on heart tissue, although we have investigated not only heart-specific mRNAs and miRNAs but also general expressed targets.

Our study has confirmed the robust stability of miRNA relative to mRNA. The observed stability is dependent on the kind of treatment and on the kind of RNA (miRNA, mRNA and ribosomal RNA). Consequently, the application of ribosomal RNA integrity as a proper representative for mRNA and miRNA integrity should be treated with caution, as it is not applicable in every case.

## Conclusions

In many studies, the putative down regulation of mRNA is considered to be due to regulatory action of miRNAs. The different stabilities of RNA classes may cause false interpretations of the observed data, especially when contaminations with RNases occur during the experiment. Therefore it is important to consider and determine the stability of the respective classes of RNA under the conditions of a given experiment. In our study we could demonstrate that after exposure to various RNases, miRNA is equally or even more stable than mRNA. This more robust stability of miRNAs supports their potential and further development as biomarkers in a wide range of applications. Due to the non-uniform stabilities of the various RNA classes under certain conditions, the application of ribosomal RNA as a representative measure of mRNA or miRNA integrity should be reconsidered.

## Methods

### Heart isolation and RNA extraction

DBA/2 mice (43 days old) from which hearts were obtained to isolate the RNA were maintained and sacrificed by cervical dislocation according to the German animal welfare law (TierSchG: § 7a, Absatz 2, Nummer 1 - 11038A4). Total RNA from the heart was isolated using peqGOLD RNAPure according to the manufacturer’s instructions (PEQLAB Biotechnologie, Erlangen, Germany).

### RNases treatment assay

Each the equal amount of total RNA from an identical RNA pool was used for the different treatments (each 2.5 μg). In a volume of 100 μl total RNA treatment was performed with indicated ribonucleases or NaOH (Table 1) at 37°C for 3 min in 1 × reaction buffer (1 mM DTT, 2 mM MgCl_2,_ 2 mM CaCl_2_, 0.9% NaCl, 50 mM Tris, pH 8.0). The ribonuclease treatment reactions were stopped by mixing thoroughly after adding one volume of Roti®-phenol/chloroform (Carl Roth, Karlsruhe, Germany). The NaOH treatment was stopped by adding the same molarity of acetic acid by vortexing.

**Table 1:** **Ribonucleases/NaOH treatments with the concentration used in this study**

***Treatment***	***Units used per ml***	***Supplier***	***Enzyme commission number***	***Literature***
RNase A	3.13	Applichem, Darmstadt, Germany	EC3.1.27.5	[36,37]
RNase H	12.50	Ambion, Kassel, Germany	EC3.1.26.4	[38]
Benzonase	8.33	Sigma, Steinheim, Germany	EC3.4.23.1	[39]
Exonuclease T	12.50	New England Biolabs, Frankfurt, Germany	EC3.1.3.1	[40-42]
Exonuclease T_7_	12.50	New England Biolabs	EC3.1.11.3	[34]
RNase I_f_	12.50	New England Biolabs	EC3.1.27.6	[31,32]
NaOH	100 μmol	Applichem		

To increase the efficiency of precipitation, a mix of 5 μg transfer RNA (Sigma) and 1.25 μg Glycogen (Carl Roth) in a volume of 25 μl was added to the reaction suspension followed by intensively mixing for 15 sec. Then the suspension was transferred to a phase Lock Gel™ light 1.5 ml tube (Eppendorf, Hamburg, Germany) and centrifuged for 15 min. Next, a volume of 90 μl of the upper phase was transferred to a new tube containing 9 μl of 3 M sodium acetate pH 4.8, mixed and incubated overnight at -20°C after adding 270 μl of 100% ethanol. The precipitate was centrifuged for 120 min, washed once with 100 μl of 70% ethanol, and resuspended in 15 μl of ribonuclease-free water. All centrifugations were performed at 44,000 × g and 4°C.

RNA quantification was determined by optical density (Nanodrop ND-1000, PEQLAB Biotechnologie). For all analysed RNAs the observed ratio 260/280 was between 1.8 and 2.0. RNA quality (integrity) was assessed using microfluidic analysis (RNA 6000 Nano kit - Agilent 2100 Bioanalyzer, both Agilent).

### Reverse Transcription quantitative Polymerase Chain Reaction (RT-qPCR)

For mRNA analysis, cDNA was reverse transcribed from 6 μl (40%) of resuspended RNA precipitate (see above) using Superscript™ II reverse transcriptase, Oligo(dT) 12-18 and random hexamer primers (all from Applied Biosystem), dNTP mix (Thermo scientific, Bonn, Germany) and DTT (Invitrogen) according to manufacturer’s instructions. To quantify the expression of hypoxanthine guanine phosphoribosyl transferase (Hprt), natriuretic peptide type A (Nppa)*,* natriuretic peptide type B (Nppb) and DNA-directed RNA polymerase II polypeptide A (Polr2A) real-time PCRs using individual TaqMan® Gene Expression Assays (Applied Biosystems) were performed in triplicates on an ABI 7300 Real-Time PCR System at 95°C for 10 min, followed by 40 cycles of 95°C for 15 sec and 60°C for 1 min. Subsequently, cDNA was mixed with TaqMan® Gene Expression Master Mix (Applied Biosystems) and the appropriate TaqMan® Gene Expression Assays (Applied Biosystems) for the gene of interest (Table 2).

**Table 2:** **TaqMan assays used in this study with the efficiency rate for each detector**

***Detector***	***Assay number***	***Efficiency%***
Hprt	Mm01545399_m1	104.7
Nppa	Mm01255747-g1	106.4
Nppb	Mm00435304-g1	102.8
Polr2a	Mm00839493_m1	94.7
miR208	TM 511	89.5
miR1	TM 2222	103.3
sno202	TM 1232	97.9
miR501	TM 1651	91.6
TaqMan® Ribosomal RNA Control Reagents	4308329	

Quantification of selected miRNAs was performed using individual TaqMan® MicroRNA Assays according to the manufacturer’s instructions (Applied Biosystems). Shortly, 6 μl (40%) of resuspended RNA precipitate (see above) were reverse transcribed using the TaqMan® MicroRNA Reverse Transcription Kit (Applied Biosystems). miR-1, miR-208, miR-501 and snoRNA202 (Table 2) were quantified in triplicates on the ABI 7300 Real-Time PCR System at 95°C for 10 min, followed by 40 cyles of 95°C for 15 sec and 60°C for 1 min.

Quantification of 18S ribosomal RNA was performed in triplicates with the TaqMan® Ribosomal RNA control reagents (Applied Biosystems) in accordance with the manufacturer’s instructions on the ABI 7300 Real-Time PCR System at 50°C for 2 min, 95°C for 10 min, followed by 40 cycles of 95°C for 15 sec and 60°C for 1 min. Assay efficiencies of detectors were determined by serial dilutions within a 4-log range of the heart total RNA and calculated with the formula 10^-1/slope^-1 (Slope = ΔCq/Δlog dilution) [21] (Table 2).

Since reference genes are also influenced by the treatments, relative quantification was performed by normalization to the untreated control. For this purpose, the Cq value of 40 cycles was assumed to represent the total digestion of RNA (0% of RNA undigested) and the Cq value of the untreated control was assigned as not digested (100% of RNA undigested). The undigested part after treatment was calculated using the following formula: x = 100 * (40 - Cq_treat_)/(40 - Cq_unt_); [x = percentage of undigested after treatment, Cq_treat_ = Cq value of treated sample, Cq_unt_ = Cq value of untreated sample].

### Statistical analyses

The data were analysed with the Prism 5.0 software (©Graphpad Software Inc., USA). The significance was calculated by the two-tailed unpaired *Student’s* t-test: *: p < 0.05; **: p < 0.01; ***: p < 0.001. SD = standard deviation.
